# Reversal of the Detrimental Effects of Post-Stroke Social Isolation by Pair-Housing is Mediated by Activation of BDNF-MAPK/ERK in Aged Mice

**DOI:** 10.1038/srep25176

**Published:** 2016-04-29

**Authors:** Rajkumar Verma, Nia M. Harris, Brett D. Friedler, Joshua Crapser, Anita R. Patel, Venugopal Venna, Louise D. McCullough

**Affiliations:** 1 Department of Neuroscience, University of Connecticut Health Center, Farmington, CT, USA; 2 Department of Neurology, University of Texas Health Science Center, Houston, TX 77030, USA.

## Abstract

Social isolation (SI) increases stroke-related mortality and morbidity in clinical populations. The detrimental effects of SI have been successfully modeled in the laboratory using young animals. Mechanistically, the negative effects of SI in young animals are primarily mediated by an enhanced inflammatory response to injury and a reduction in neurotrophic factors. However, the response to brain injury differs considerably in the aged. Given that SI is more prevalent in aged populations, we hypothesized that isolation, even when initiated after stroke, would delay recovery in aged mice. We found that aged isolated male mice had significantly increased infarct volume, neurological deficits, and serum IL-6 levels three days after stroke compared to pair housed (PH) mice. Using RT^2^ Profiler PCR Array and real-time quantitative PCR we found several important synaptic plasticity genes were differentially expressed in post-stroke SI mice. Furthermore, paired mice showed improved memory and neurobehavioral recovery four weeks after injury. Mechanistic and histological studies showed that the beneficial effects of pair housing are partially mediated by BDNF via downstream MAPK/ERK signaling and restoration of axonal basic myelin protein levels.

Ischemic stroke is the fifth leading cause of death in the United States^1^ and the number one cause of adult disability. Nearly three-quarters of all strokes occur in the elderly^2^. Recent studies using aged animals have revealed important age differences that are relevant to our understanding of the acute cellular responses to stroke and for the identification and development of potentially beneficial interventions^3^. In addition, the recovery phase after an acute ischemic stroke is markedly slower and incomplete in aged animals^4^,^5^. These age-associated behavioral impairments result from region-specific changes in dendritic morphology, cellular connectivity, Ca2+ dysregulation, altered gene expression, inflammation and other factors that affect brain plasticity and cognition^6^,^7^,^8^. Given that the responses of the central nervous system to stroke are age dependent^9^,^10^ pre-clinical stroke studies utilizing young animals may not adequately assess age specific pathological changes after cerebral ischemia.

In addition to the age-specific inflammatory and pathophysiological changes following stroke, the elderly have higher rates of social isolation than their younger counterparts^11^. Social isolation, loneliness, and psychosocial distress have been linked to increased stroke incidence^12^,^13^,^14^,^15^,^16^ and mortality following the index event^11^,^17^,^18^. Long-term social isolation disrupts myelin structure in juvenile mice^19^ and leads to cognitive impairment in adulthood^20^, even without injury. Intact myelin health is critical for learning and memory^21^ and also can influence brain plasticity^22^. Recent work has shown that SI reduces BDNF expression in young mice, which contributes to post-stroke depressive phenotypes and impaired post-stroke cognitive recovery in isolated animals^23^. Under normal conditions, BDNF, a well-established neuro-protective and neuro-restorative protein, provides trophic support to injured neurons by activating pro-survival pathways. Enhanced BDNF expression aids tissue repair after stroke in young animals^23^,^24^. However, given that BDNF levels and neuronal plasticity decline with aging in both humans and rodents^25^, the mechanisms underlying the beneficial effects of pair housing may differ in the aged brain. Affiliative social interactions increase neuronal plasticity and enhance functional recovery after stroke possibly through enhanced BDNF signaling, increased expression of oxytocin^26^ and a reduction in pro-inflammatory cytokines such as IL-6^27^, and other targets that have not been validated in aged animal^28^,^29^. Therefore, it is imperative to examine the effects of SI in aged animals to appropriately model the population at risk. This is an essential step if we hope to develop efficacious therapies for people at highest risk for stroke, the elderly. In this work we first determined if SI increased infarct size in aged animals, then evaluated if SI enhanced mortality and slowed behavioral recovery, and finally assessed if the detrimental effects of SI could be reversed by an affiliative housing and investigated possible mechanisms with a focus on plasticity. The effects of SI on genes involved in synaptic plasticity, including immediate early genes (IEGs) and other genes important for long-term potentiation (LTP) and long-term depression (LTD) in addition to genes important for synapse remodeling was evaluated in aged animals. In this study both acute and chronic endpoints were evaluated.

## Results

### Survival/mortality after stroke

**Figure 1:**
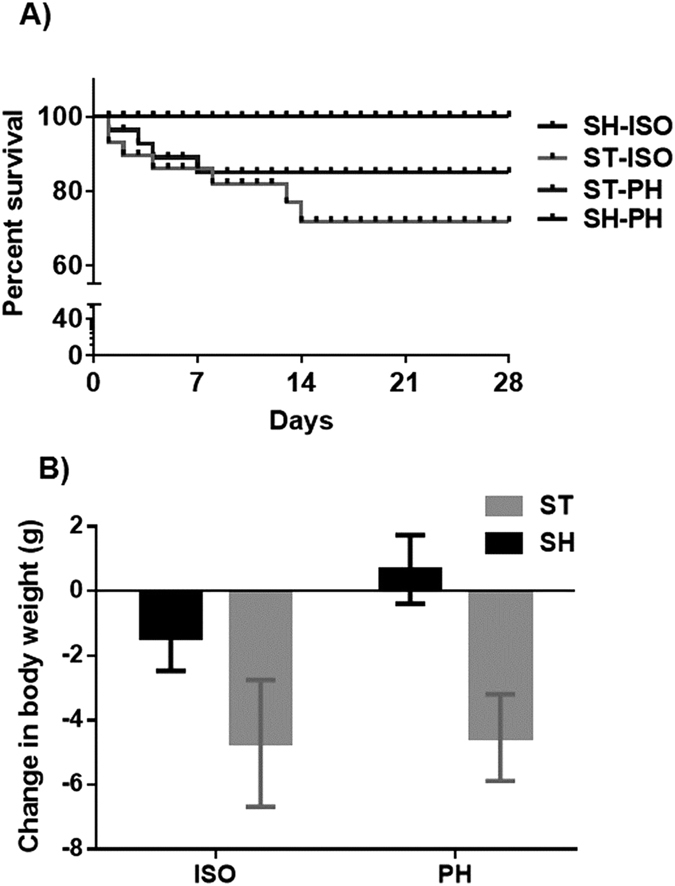
(**A**) Kaplan–Meier survival curve in aged mice four weeks after stroke or sham surgery. (**B**) Change in body weight as measured prior to stroke and at the time of sacrifice. There was a significant effect of stroke (p < 0.05) but no effect of housing.

In our acute cohort (euthanized 72 hours after stroke) one ST-PH (stroke paired with a sham mouse) mouse died while two ST-ISO (mice subjected to both stroke and isolation) died during the three day survival period, leading to a mortality rate of 7.1% in ST-PH vs. 14.2% in ST-ISO mice. In a chronic survival cohort (collapsed to include animals used for both behavioral and western blot analysis) euthanized 30 days after stroke, eight ST-ISO and four ST-PH mice died over the 30 day survival period for a total loss of 16 mice (12 deaths and 4 mice that were not used due to their loss of partner in the ST-PH group). Higher mortality was seen in the ST-ISO mice (ST-PH 19.04% vs. 28.5% in ST-ISO group). No deaths occurred in sham mice in either cohort (Fig. 1).

### Changes in body and spleen weight

There was a significant main effect of stroke on body weight, however, no effect of housing or interaction between stroke and housing conditions was seen (Fig. 1b). No changes in spleen weights were observed in any of the groups (data not shown).

### Effect of pair housing on infarction and tissue atrophy

**Figure 2:**
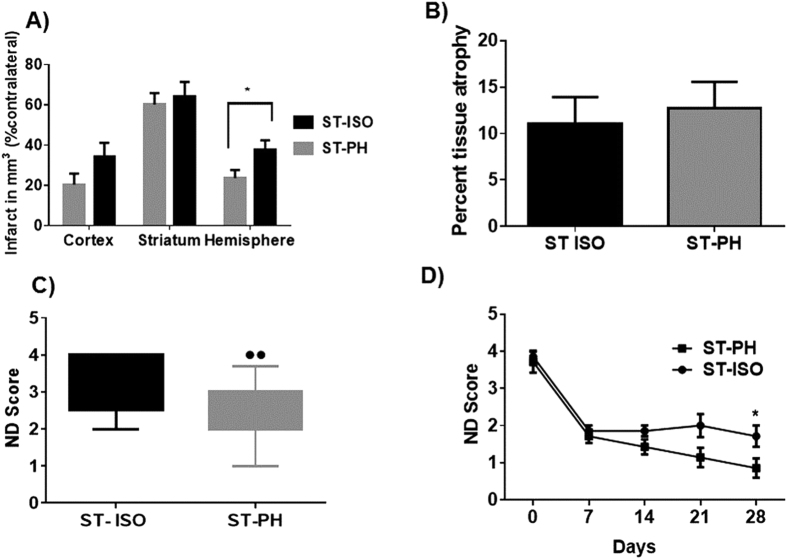
Effect of stroke on infarct volume and neurological deficit score (NDS) in socially isolated (ST- ISO) and pair-housed (ST-PH) mice. (**A**) A significant difference (p < 0.05) was found in total hemispheric infarct between ST-ISO and ST-PH mice (37.7 ± 4.73 vs 20.0 ± 6.4) in mice sacrificed 72 hours after reperfusion. (**B**) Effect of housing condition on brain tissue atrophy. No changes in % tissue atrophy were observed between ST-ISO and ST-PH group after 4 weeks. (**C**) Congruent to infarct volume changes, ST-PH group had a significant improvement in behavioral recovery compared to ST-ISO mice 72 hours after stroke. (**D**) However, unlike tissue atrophy, deficits in the NDS were significantly different (p < 0.05) between ST-ISO and ST-PH group at day 28. The ST-PH mice showed progressive recovery while ST ISO mice did not recover further after the initial acute recovery during week 1.

ST-ISO mice had a significantly larger infarct size (p < 0.05) compared to ST-PH mice (37.7 ± 4.7 vs 20.0 ± 6.4) in the acute survival cohort (Fig. 2a). However, no significant difference was observed in tissue atrophy between ST-ISO and ST-PH mice in the chronic survival group (Fig. 2b).

### PH mice have improved recovery

ST-PH mice had significantly reduced neurological deficit scores (NDS) when assessed three days after stroke versus the ST-ISO mice (p < 0.05) (Fig. 2c). In the chronic survival cohort, both ST-ISO and ST-PH groups had equivalent but incomplete recovery at day 7. Importantly, after 7 days, the ST-ISO group showed no further behavioral recovery, but ST-PH mice continued to improve until the time of sacrifice leading to a significant difference in the NDS at day 30 between SI and PH mice (p < 0.05) (Fig. 2d).

### Sensorimotor deficits are improved by pair housing

**Figure 3:**
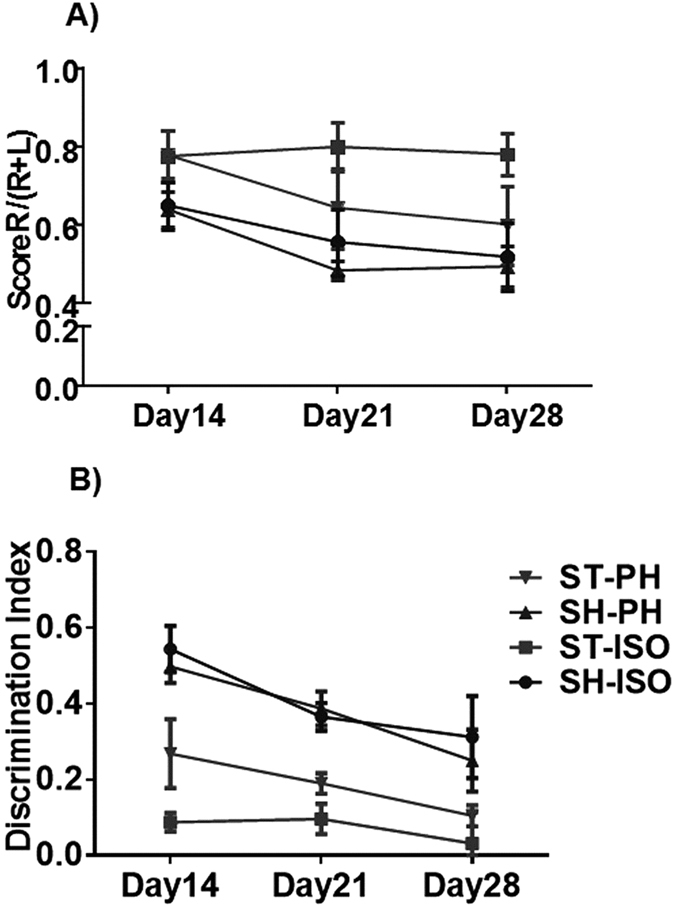
Show the analysis of sensory-motor deficits and impaired memory in chronic survival cohorts 3 (**A**) Enhanced sensory-motor deficits were seen in isolated mice after chronic survival in the corner test. Scores are presented as the ratio of turns towards the non-impaired side [R/(R + L)]. Both SH-PH and SH-ISO remained at approximately 0.5 ± 1, with nearly equivalent turns to the right and left (normal). ST-PH mice showed progressive recovery while ST-ISO did not recover (p < 0.05) even after 4 weeks. (**B**) NORT demonstrated impaired memory in SI mice. A significant interaction [F (6, 84) = 8.042, P < 0.001] in discrimination index was observed between housing and stroke condition. Moreover, there is a significant main effect (p < 0.001) of both stroke and housing condition was seen in the NORT.

Aged stroke mice were given at least 10 days to recover prior to any structured behavioral testing. Mice were assessed at days 14, 21 and 28. No significant interaction was observed between stroke and housing conditions using repeated measures two-way ANOVA. However, a significant main effect of stroke was observed, even 4 weeks after injury, demonstrating the chronic persistence of stroke-related deficits in all aged mice (p < 0.01). Progressive recovery was seen in ST-PH mice compared to the ST-ISO cohort and scores were significantly different between groups (p < 0.05, t-test at day 28 ST-ISO vs ST-PH) (Fig. 3a).

### Pair housing improved learning and memory deficits

Mice were assessed on the NORT two weeks after MCAO. A significant interaction [F (6, 84) = 8.042, P < 0.001] between housing condition and stroke was seen on the initial NORT assessment. However, these differences were lost with repeated testing at week 3 and 4 (Fig. 3b).

### Mouse Synaptic plasticity genes expression in social isolation

A total of 84 key mouse synaptic plasticity genes were analyzed using a gene expression profile in the ipsilateral frontal perilesional cortex from brain homogenates. A two-way ANOVA analysis evaluated the effect of housing, stroke and the interaction between these factors. Out of the 84 genes investigated, a significant main effect of stroke was found ion 13 genes (≥2 fold and p < 0.05). Among those, 4 genes were down-regulated and 9 were upregulated after stroke. Only 9 genes were modulated as a consequence of housing as the main effect. A significant interaction of housing and stroke was found in 6 genes (table 1).

### Serum IL-6 levels and BDNF immunoassay

**Figure 4:**
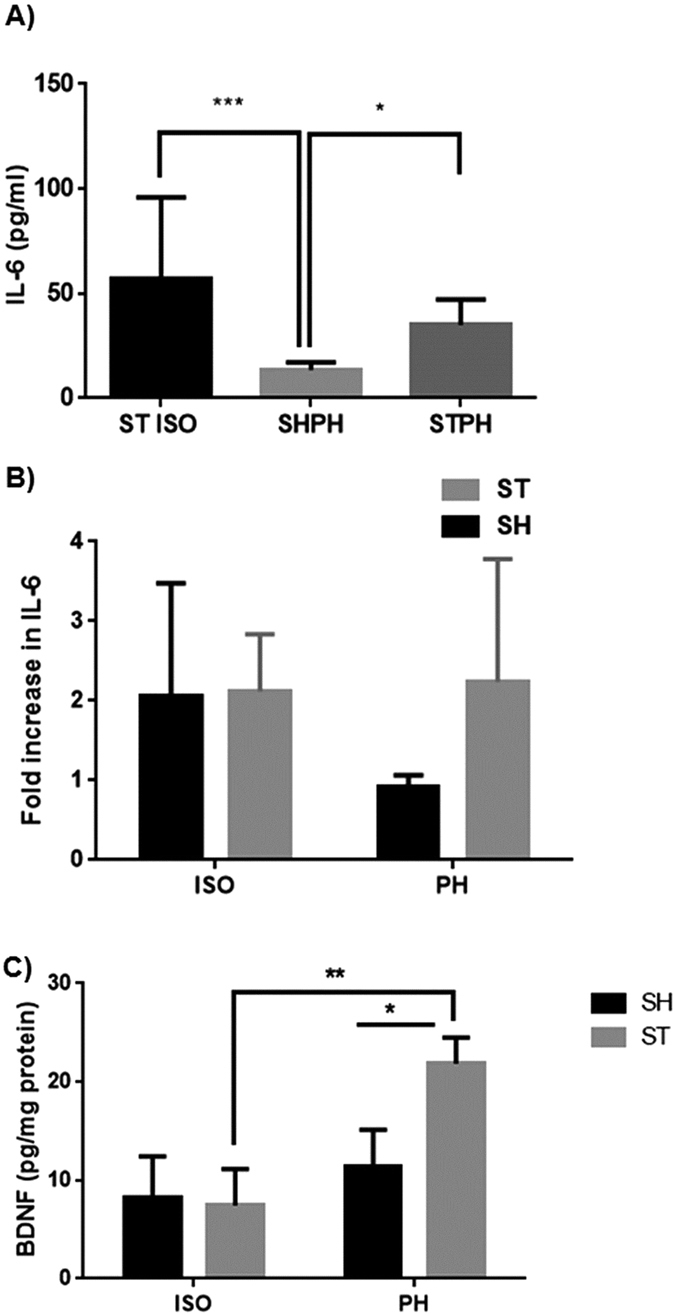
(**A**) Plasma IL-6 levels. ST-ISO had significantly higher (P < 0.05) IL-6 levels as compared to the ST-PH in the acute survival cohort. (**B**) In the chronic survival group there was no main effect of SI or any interaction [(F (1, 17) = 0.2678; P = 0.6115] between stroke and housing condition. (**C**) Total BDNF expression and activity was higher in PH animals and significant interaction [F 1, 8 = 7.13 P = 0.0238] between housing and surgery (stroke) was found in mice.

A two-fold increase in plasma IL-6 levels was seen 72 hours after stroke (Fig. 4a) in the ST-ISO group compared to ST-PH group (P < 0.05). No main effect of stroke or an interaction between stroke and housing condition was seen at 4 weeks [(F (1, 17) = 0.2678; P = 0.6115] (Fig. 4b). Total BDNF expression was similar to what was observed in western blot experiments. A significant main effect of stroke and housing, as well as an interaction [F 1, 8 = 7.13 P = 0.0238] between stroke and housing condition, was seen at 4 weeks (Fig. 4c)

### Expression of MBP and BDNF after chronic survival

**Figure 5:**
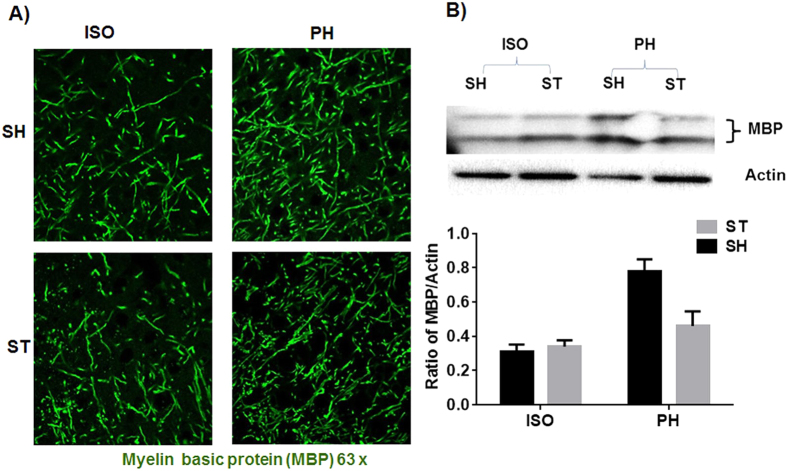
(**A**) Representative image of a mouse brain section (63× magnifications) showing MBP expression at 4 weeks after MCAO in the penumbral region. Immunostaining with MBP showed that myelin density is lower in the SI group vs PH cohorts. (**B**) Western blot analysis confirmed that SI decreased (p < 0.05) MBP expression.

**Figure 6:**
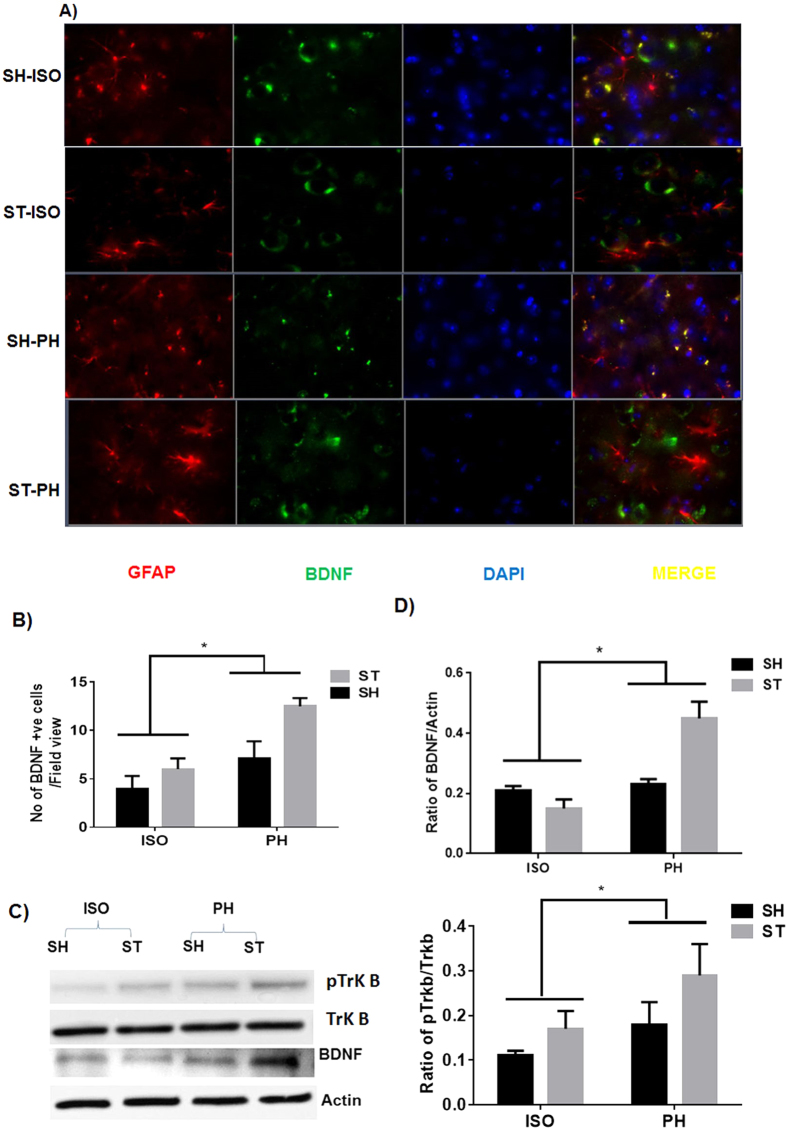
(**A**) BDNF expression after stroke. Immunofluorescent staining for GFAP (red), BDNF (green) and DAPI (blue) in the brains 4 weeks after MCAO (representative of 4 animals). Increased expression of BDNF was found in ST-PH group as compared to other groups. (**B**) A significant main effect of housing was found in the average number of BDNF + neurons (P < 0.05). (**C**) This was also confirmed by western blot.

**Figure 7:**
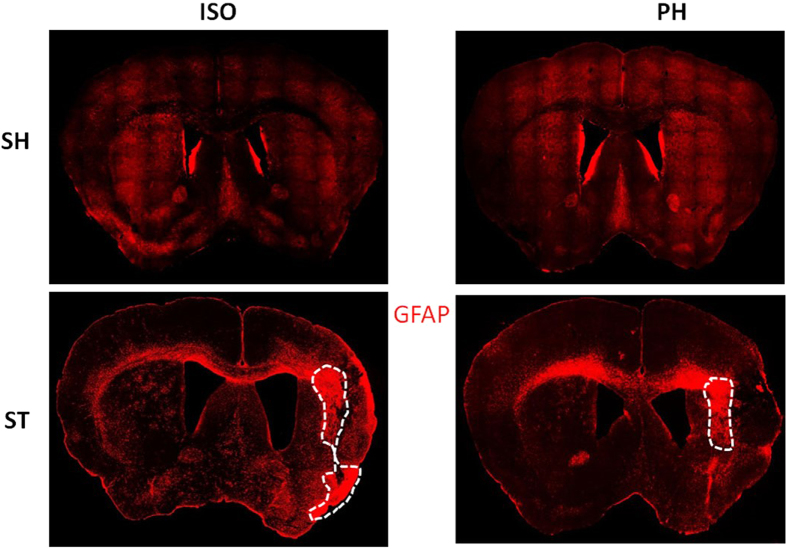
A representative mosaic image of a mouse brain section (10 × magnifications) for GFAP expression 4 weeks after MCAO. Immunostaining with GFAP (red) showed that GFAP + cells were activated at the site of infarction in both stroke groups. Dotted lines show that dense GFAP expression (the glial scar) was confined to the striatal region in ST-PH mice while it was larger and spread to both the striatum and cortex (n = 4 per group) in ST-ISO mice.

**Figure 8:**
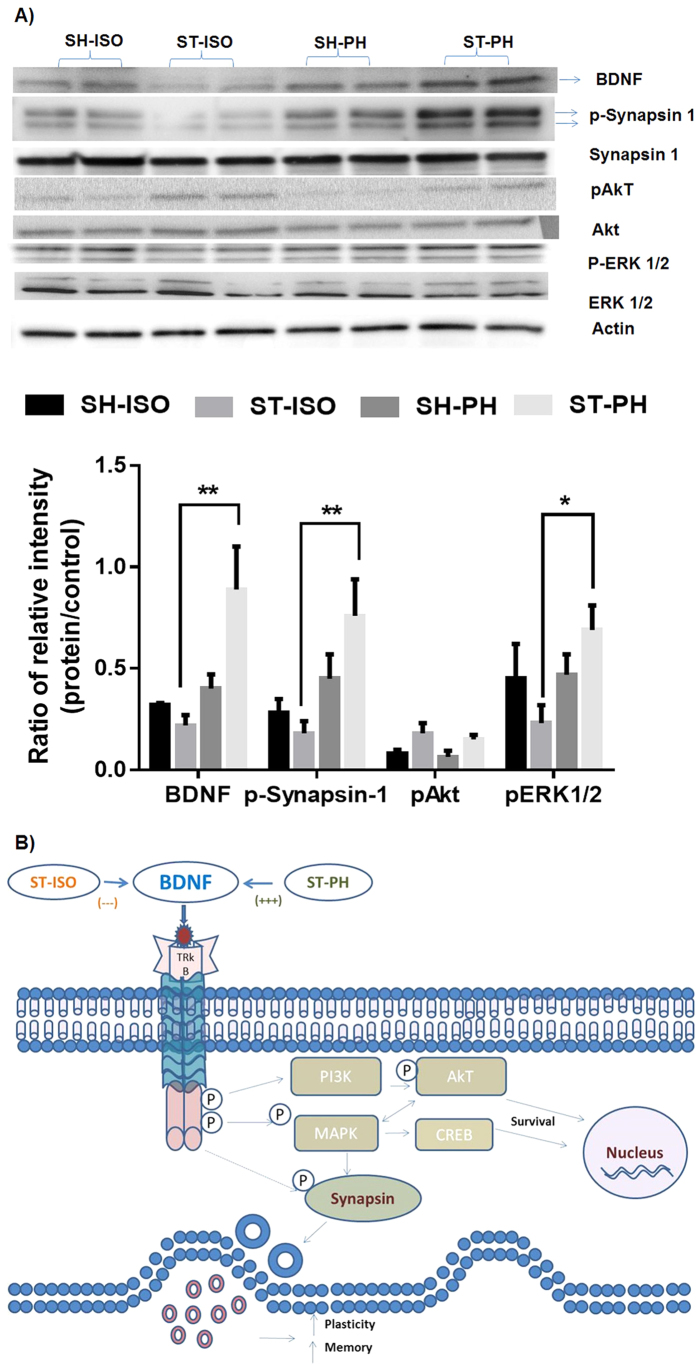
(**A**) Activation of BDNF-MAPK-synapsin pathways in pair-housed mice after stroke. Brain tissue homogenates 4 weeks after pair housing showed an up-regulation of BDNF in ST-PH mice. Western blot analysis shows upregulation of p-synapsin and pERK1/2 in ST-PH mice as compared to ST-ISO mice. pAkt levels were elevated in both stroke groups. (**B**) Schematic illustration of the potential mechanism by which pair housing, BDNF, TrK-B receptor and the downstream signaling pathways could enhance neurobehavioral recovery. SI decreases BDNF levels in the brain. BDNF after binding to its receptor can activate MAPK via phosphorylation. Activation of MAPK leads to enhanced phosphorylation and activation of synapsin-1 which increases the synaptic release of neurotransmitters such as glutamate and GABA, increasing synaptic transmission, LTP, memory, cognition and recovery after stroke.

MBP expression was significantly higher in the PH group [ST-PH and SH-PH (sham mice paired with a stroke partner)] compared to SI mice [ST-ISO and SH-ISO (stroke and sham isolated mice)]. This was confirmed by both IHC and western blot (P < 0.05) (Fig. 5a,b) suggesting that MBP expression is reduced after isolation and this effect is independent of stroke. An increased number of BDNF positive cells (P < 0.05) were found in ST-PH mice compared to ST-ISO mice (Fig. 6a,b). There was a significant interaction [F (1, 19): P = 0.0423] between housing conditions and stroke in BDNF expression, with significantly higher BDNF expression in ST-PH (P < 0.01 t-test between ST-PH vs. STISO) compared to ST-ISO mice (Fig. 6c). The corresponding increase in pTrkb expression further suggests that there is an increase in BDNF activity in ST-PH group (Fig. 6C,D). Importantly, GFAP expression was higher and extended to cortical regions in ST-ISO group (Fig. 7). To evaluate potential mechanisms by which BDNF could mediate behavioral recovery in PH stroke mice, we examined brain expression of PI3K/AKT, a downstream pathway induced by BDNF signaling. Increased phosphorylation of AKT after stroke was seen in both stroke groups (ST-ISO and ST-PH) to an equivalent degree. However assessment of MAPK/ERK1/2, a distinct pathway involved in BDNF signaling, showed that pERK1/2 expression was increased in ST-PH mice compared to ST-ISO cohorts. Similarly, increased phosphorylation of synapsin-1 was found in the ST-PH group (Fig. 8a). This suggests that BDNF may increase synaptic function either directly by increasing phosphorylation of synapsin-1 or indirectly via MAPK activation (Fig. 8b).

## Discussion

Age is the most important non-modifiable risk factor for stroke. Recovery is slower and incomplete in aged animals^5^ even with smaller infarct volumes^4^. Comorbidities such as hypertension, obesity, diabetes, dyslipidemia and increased systemic inflammation increase the probability of poor outcomes in older patient^18^,^30^,^31^. Recent reports suggest that both social isolation and feelings of loneliness can increase the risk of illness and death in humans^11^,^18^. Importantly, the number of stroke patients exposed to social isolation is growing rapidly, especially in the elderly who have decreased economic resources, impairments in mobility, and have often lost their contemporaries and spouses, all of which limit social contact^11^. These individuals are also at increased risk for the development of cardiovascular diseases^32^, infections^33^, cognitive deterioration^34^, and increased mortality^11^. Aging produces many cognitive, emotional, and neural changes that impact social perception and the response to affiliation and isolation changes drastically in the aged^35^. Therefore, the mechanisms that are involved in the detrimental effects of isolation in young animals or patients may not be relevant to aged subjects.

In this work, we systemically assessed the effects of social isolation on aged animals after stroke on infarct damage, mortality, gene expression, behavioral recovery and neuroprotective signaling cascades. Aged mice isolated immediately after stroke (ST-ISO) had increased infarct damage and poorer behavioral recovery compared to pair-housed stroke (ST-PH) mice consistent with previous work performed in young animals^29^. However, unlike what is seen in isolated young mice that have continued tissue loss and significant progressive tissue atrophy after chronic survival, no difference in brain atrophy was seen four weeks after stroke in aged mice^27^. This may be due to the overall reduction in stroke volume in aged mice^4^, the incomplete recovery seen in aged mice after stroke^5^ or other factors related to the distinct morphology of neuronal damage seen in the aged brain^7^. Other investigators have found increased tissue damage in aged rodents early after stroke both in MCAo^36^ and distal model of stroke^37^; although in our models (reperfusion) we have repeatedly shown that aged male mice have smaller infarcts than young male mice^4^, although they have more severe behavioral impairments. It is important to note that in this work we did not directly compare aged and young mice. Despite equivalent histological damage between the groups, behavioral recovery remained poor and incomplete in aged mice that were isolated after stroke, suggesting that the effects of SI are independent of histological damage and may be due to more subtle deficits in connectivity or repair. We found that inflammatory cascades initiate more swiftly and aggressively in isolated mice compared to mice that are pair housed after injury. These effects become attenuated over time as shown by analysis of plasma IL-6 levels, which were significantly elevated during the acute phase, but were equivalent between the paired and isolated groups after 4 weeks. Moreover, early neuro-inflammatory events are major contributors to cellular damage and mortality after stroke^38^ but later events such as enhancements in BDNF and myelin protein integrity may be responsible for chronic functional recovery^39^,^40^. One important caveat is that measurement of brain atrophy using cresyl violet (CV) staining may be a less sensitive measure of histological damage particularly at chronic time points, as it is difficult to assess the specific type of cells lost (i.e., neurons vs. astrocytes). Interestingly, unlike what was seen by CV staining, analysis of the glial scar suggests that SI mice show delayed recovery due to astrogliosis. Although, the formation of an early glial scar may prevent progression of the infarct, this may also hamper brain repair by reducing neurogenesis or axonal re-growth^41^. The enhanced cortical glial scarring seen in ST-ISO mice could have contributed to failed axonal regeneration via formation of a physical barrier^42^ and inhibition of remyelination^43^. This is consistent with our finding that SI led to a reduction in myelin content. Moreover, reactive gliosis and scar formation have been shown to enhance ischemia-induced behavioral deficits^44^.

BDNF is an important neurotrophic factor which is required to maintain synaptic plasticity in the adult brain^45^. We found that isolation induced decreases in BDNF levels that were restored by pair housing in aged mice, supporting the hypothesis that PH restored trophic support and leads to an acceleration of neuronal recovery. Although it is not clear whether PH prevented the stroke-induced loss of BDNF (changes are not seen in sham animals regardless of housing conditions, see Fig. 8a) or increased BDNF expression during recovery, our data suggests that PH not only maintains BDNF levels, but also potentiates its expression and activity as suggested by increased pTrkb expression. Aging as well as social isolation decrease BDNF expression^23^,^46^,^47^. Recent studies have shown that BDNF treatment enhances post-stroke functional recovery in aged mice^39^ implying that loss of BDNF is detrimental. Restoration of BDNF via affiliative social interactions may not only enhance recovery, but may also help alleviate other common post-stroke disorders such as depression.

We have shown that several important genes involved in the maintenance of LTP and LTD (eg. *BDNF CamK2a, GABRA5, AMPK 2, GRIN2b and GRIN2c, IGF1 and MAPK1;* Table 1) were differentially expressed in SI animals. These genes play an important role in post-stroke recovery. Among the many genes that were altered, we focused on BDNF as it has been identified as having a major role in synaptic plasticity. Our work suggests that PH induces BDNF, which facilitates behavioral recovery via increased phosphorylation of the synaptic vesicle protein, synapsin-1, via MAPK signaling. The increased phosphorylation of synapsin-1 leads to the release of both glutamate and GABA^48^, which are involved in maintaining synaptic plasticity, learning and memory. Further studies are required to dissect out the contribution of each gene in post-stroke SI-induced deficit. Previous studies have shown that GABAergic mechanisms mediate changes in neuronal excitability and have a central role in the functional recovery of the peri-infarct cortex after stroke^49^. Therefore, it might be possible that the incomplete recovery seen in these aged mice after stroke is due in part to altered GABAergic transmission. These findings are consistent with previous reports that show that pharmacological and genetic knockdown of α5-GABA_A_ receptors (*GABRA5*) enhances long-term potentiation and improves performance on learning and memory tasks^49^,^50^. No prior study has shown the effect of above mentioned LTP related gene either alone or in combination on post-stroke SI in aged mice but, the effect of some of these gene (e.g AMPK) on the action of BDNF in stroke as well as other disorders have been described^51^. In addition to GABRA5, Other genes like Nf-kb1 and Rela which play an important role in learning and memory^52^, were reduced in PH mice. Loss of the Nf-kb1 and Rela genes abolishes the detrimental effect of social isolation in young male mice^29^. No changes were found in Akt between SI and PH stroke mice suggesting that the detrimental effects are specifically linked to MAPK signaling.

One other very novel finding from this work is that both SI and stroke decreased the expression of myelin basic protein (MBP). Our data suggest that the effects of isolation are even more detrimental than the effect of stroke on MBP expression. This is consistent with previous research showing that the absence of social stimuli leads to significant hypomyelination in the prefrontal cortex of in young mice^19^. Our data demonstrate that pair-housing prevents myelin loss, which may improve learning and memory after stroke by maintaining connectivity or enhancing conduction/repair in damaged areas. This is further supported by recent work that shows that BDNF also improves functional outcome after stroke by mediating axonal growth, OPC proliferation, oligodendrocyte differentiation, remyelination, and fiber tract connectivity^53^. Both *in vitro* and BDNF knockout studies have demonstrated that BDNF directly promotes the proliferation and differentiation of oligodendrocyte precursor cells (OPC) and myelination^54^,^55^. Moreover, very recent work demonstrated that BDNF treatment leads to a significant increase in the number of proliferating cells, including OPCs, after white matter injury as improved functional recovery after stroke. The SI-induced loss of object recognition memory can be partially explained by dysfunction of perirhynal cortex; the cortical region involved in objective recognition memory^56^, as suggested by dense glial scarring in this region in SI mice. Taken together, the present study suggests that the beneficial effects of pair housing on neurobehavioral recovery are the result of multiple mechanisms including a reduction in acute inflammation, chronic elevation of BDNF expression, and prevention of myelin loss. Social isolation exacerbates the neuropathological as well as the neurobehavioral consequences of stroke in aged animals. This is the first study to evaluate the effects of social isolation in aged animals. We show that isolation increases infarct damage and impairs functional recovery after stroke in aged mice. Impaired behavioral recovery is linked to an enhanced acute inflammatory response, changes in various genes that control LTP and LTD, reductions in BDNF levels, enhanced glial scar formation and enhanced myelin loss. Supportive social contacts may prevent the detrimental effects of SI after stroke. As many elderly stroke patients are at risk for social isolation, potential mechanisms need to be explored so that effective therapeutic strategies can be developed.

## Methods

### Experimental animals

All animal protocols were approved by the University’s Institutional Animal Care and Use Committee at the University of Connecticut Health Center and were performed in accordance with National Institutes of Health guidelines. Aged C57Bl/6 male mice (1618 months; 40 ± 2 g) were purchased from National Institute on Aging (NIA), Bethesda USA. After arrival, the mice were acclimatized in the animal care facility for at least two months and were maintained at an ambient temperature and humidity controlled vivarium with free access to food and water ad libitum. A total of 140 aged mice were randomly pair housed (2 mice per cage) for three weeks. During pair housing all mice were examined daily for compatibility (e.g., weight gain and the absence of fight wounds). A total of seven pairs of mice (14 mice) were excluded prior to any manipulation due to incompatibility. After three weeks of pair housing, 126 mice (63 pairs) were randomly assigned to stroke or sham surgery. Immediately after surgery, mice were randomly assigned to one of four groups using a two-way factorial design. Surgical condition (sham (SH) versus stroke (ST)) was the first between-subjects factor and housing condition (housed with sham (SH), housed with stroke (ST) or housed in isolation (ISO), was the second between-subjects factor. The following groups were generated; Sham Isolated (SH ISO), Sham paired with Stroke (SH-PH), Stroke paired with Sham (ST-PH), and Stroke Isolated (ST-ISO). The assigned housing conditions were maintained throughout the reperfusion period (72 hours, 15 days4 weeks depending on the cohort) until sacrifice. If either mouse among the pair died, the partners were excluded from the study.

Experiments were conducted in two separate cohorts. A total of 140 aged (70 compatible pairs) mice were divided into three different cohorts that produced an “acute” (total 42 mice or 21 pairs), a “sub-chronic” (12 mice or 6 pairs) and a “chronic” survival cohort (84 mice or 42 pairs). The first experimental cohort was an acute survival group [with a total of 38 surviving mice: ST-PH = 13, SH-PH = 13 and ST ISO = 12] used for histological assessment, acute neurological deficit analysis, and for measurement of serum IL-6 levels 72 hours after reperfusion. In a second experimental cohort (15 days survival n = 3 mice/group) the frontal ipsilateral cortex was utilized for gene analysis. In the third experimental cohort (“chronic survival”) [Total 47 surviving mice: ST ISO = 14, SH ISO = 9, ST-PH = 12 and SH-PH = 12] mice were assessed for behavioral deficits weekly (Supplementary Fig. 2). This cohort was also used to assess tissue atrophy, glial scar formation, and other chronic immunohistochemical analyses. An additional cohort of chronic survival animals [for a total of 21 additional mice: ST-ISO = 6, SHISO = 5, ST-PH = 5 and SH-PH = 5] was used for western blot analysis. All behavioral tests were performed by investigators blinded to housing conditions.

### Stroke model

Focal transient cerebral ischemia was induced by a 60 minute right middle cerebral artery occlusion (MCAO) under Isoflurane anesthesia followed by reperfusion and survival either for 72 hours (acute cohort), 15 days (RNA cohort) or 4 weeks (chronic cohorts) as described previously^27^. In sham mice, the identical surgery was performed except the suture was not advanced into the internal carotid artery. All animals were fed with wet mash for one week after surgery to ensure adequate nutrition as animals have rearing deficits after stroke. Additionally, a daily subcutaneous injection of normal saline (volume = 1% v/w) was given to all animals in all housing groups for one week.

### Cresyl violet (CV) staining for infarct volume and tissue atrophy analysis

All animals in the acute cohort were sacrificed at 72 hours after stroke and mice in the chronic survival cohort were sacrificed four weeks after stroke/sham surgery with an overdose of Avertin (250 mg/kg i.p). The mice were further processed for CV staining as in^29^ and infarct volumes were quantified from digitalized section images using Sigma Scan Pro software as in^57^. Similarly, mice in the chronic survival cohort were used for calculation of tissue atrophy. Data analysis was performed by an investigator blinded to housing conditions^27^.

### Corner Test

The corner test assesses sensory and motor deficits following brain injury^58^. In this test, two pieces of cardboard were moved together to form a progressive angle of approximately 30 degrees in front of the nose. Contact with the vibrissae leads to rearing. The percentage of right turns was calculated over twenty trials.

### Novel Object Recognition Task (NORT)

The NORT is used to evaluate cognition and recognition memory in rodent models^59^. This test is based on the tendency of mice to spend more time exploring a novel object than a familiar one. This preference is a measure of intact recognition memory. During habituation animals were allowed to explore an empty arena for at least 10 min. Twenty-four hours after habituation; the animals were exposed to the familiar arena with two identical objects placed at an equal distance for 10 min. If the total time of exploration of these objects is >20s these mice qualify for the experimental trial^60^. The next day, mice explored the open field in the presence of the familiar object and a novel object. The NORT was performed weekly starting at day 14 after stroke and new objects were used every week. The time spent exploring each object was recorded and the discrimination index (DI) is calculated by using the formula DI = (TN−) F)/(TN+) ), where Tn = time spent exploring the novel object and Tf = time spent in exploring of familiar objects.

### Mouse Synaptic Plasticity RT2 Profile PCR Array

Total RNA was isolated with Qiagen’s RNA isolation kit from the frontal cortical tissue of the ipsilateral (stroke) hemisphere. An equal amount of RNA was then converted into cDNA using SA Biosciences’s RT2 First Strand Kit (Cat # 330401) as per manufacturer’s protocol. Synaptic plasticity gene profiling was done using a 96 well format RT^2^ Profile PCR Array Mouse Synaptic Plasticity kit (SABiosciences, Cat # PAMM-126Z) with a Bio-Rad CFX 96 qRT-PCR instrument. This array analyzed 84 genes involved in mouse synaptic plasticity, including immediate-early response (n = 30) and late response genes (n = 2), genes involved in long-term potentiation (LTP) (n = 28), long-term depression (LTD) (n = 21), cell adhesion (n = 9), extracellular matrix and proteolytic processing (n = 5), CREB cofactors (n = 10), neuronal receptors (n = 19), postsynaptic density genes (n = 15), as well as other genes involved in synaptic plasticity (n = 2). The relative abundance of each mRNA species was assessed following the manufacture’s recommendations. The data was analyzed using programs provided by the manufacturer and individual genes were analyzed using prism software. In the expression studies, a gene was considered differentially regulated if the difference was ≥2 fold and p values were <0.05 compared to control. A total of twelve animals were utilized for this experiment (n = 3/group)

### Immunohistochemistry (IHC)

IHC staining was performed on 30-μm sections mounted on Fischer Scientific Superfrost Plus charged slides in the brain of chronically survived cohort of mice using the standard protocol as described previously^27^. BDNF, MBP and GFAP antigen were probed using their specific antibody [BDNF 1:500 (BD bioscience); Myelin basic protein (MBP) 1:1000 (Abcam) or GFAP-Cy3 1:500 (Sigma)]. Three coronal brain sections per mouse (n = 4 per group), taken 0.02, 0.45, and 0.98 mm from bregma, were stained and visualized for quantification at 20×/63× magnification at the core/penumbra junction (Supplementary Fig. 1). A blinded observer quantified BDNF and DAPI positive cells using Image J software (NIH). The average numbers of cells visualized from 3 adjacent regions at the core/penumbra junction were recorded for each mouse. Confocal microscopy (LSM710 Metaconfocal laser scanning microscope, Carl Zeiss Micro-Imaging) was performed to visualize MBP on brain tissue sections.

### Whole cell lysis and western blot

Brain samples were obtained 4 weeks after stroke and were immediately dissected into the right (R; ischemic) and left (L; non-ischemic) hemispheres. Tissue was homogenized with Dounce homogenizers in cold triton lysis as previously described^27^. A total 30μg of protein was loaded in each well and resolved on 4%–15% SDS gels and transferred to a PVDF (polyvinylidene difluoride) membrane. Total myelin, BDNF, pTrkb, Trkb synapsin, p-synapsin, Akt, p-Akt P-MAPK (ERK 1/2), MAPK and actin were detected by the following antibodies; Myelin Basic Protein (1:1000 Abcam); BDNF (1:1000 EMD millipore ); pTrkB 1:500 (706, Santa Cruz), TrkB 1:1000 (Santa Cruz); Synapsin and p-synapsin (1:1000, CST); Akt and p-Akt (1:1000, CST); ERK1/2 and p-ERK1/2 (1:2500 CST) and actin (1:5000, Sigma) respectively. All blots were blocked with 5% non-fat dry milk or 4% BSA for 1h at room temperature and incubated overnight with primary antibodies at 4 °C. Secondary antibodies were either goat anti-rabbit IgG 1:10,000 (GE Healthcare Life sciences) or goat anti-mouse IgG 1:10,000 (GE Healthcare Life sciences). Thermo Scientific Super Signal West Pico Chemiluminescent Substrate was used for signal detection. Densitometry (n = 5-6 per group) was performed with Image J software.

### Measurement of serum IL-6 and BDNF immunoassay in brain tissue homogenates

Serum Interleukin-6 (IL-6) levels were assessed at sacrifice (72 hours and 4 weeks) in both cohorts of mice according to manufacturer’s instructions (eBiosciences, San Diego, CA). Total BDNF was measure by using BDNF Emax^®^ ImmunoAssay System by Promega following their protocol.

### Spleen and body weight

Body weight was recorded daily for the first seven days after stroke and then weekly until sacrifice. The spleen was removed after perfusion. The splenic weight at sacrifice was also recorded and represented as a fraction of spleen weight over body weight multiplied by 100.

### Statistics

All data were analyzed and expressed as means ± S. E. M. Behavioral and other tests comprising three or more groups were analyzed by one-way ANOVA with a Bonferroni post hoc test to correct for multiple comparisons or Student’s t-test except for neurologic deficit scores, which was analyzed by Mann–Whitney U test. In a two-factor interaction ‘group versus days’ a two-way ANOVA with repeated measures was used followed by a Bonferroni post hoc test. A probability value of p < 0.05 was considered to be statistically significant. Investigators performing and analyzing behavioral tests and other analysis were blinded to housing and surgical conditions.

## Additional Information

**How to cite this article**: Verma, R. *et al.* Reversal of Detrimental Effects of Post-Stroke Social Isolation by Pair-Housing is Mediated by Activation of BDNF-MAPK/ERK in Aged Mice. *Sci. Rep.* **6**, 25176; doi: 10.1038/srep25176 (2016).

## Supplementary Material

Supplementary Information

## Figures and Tables

**Table 1:** List of genes showing either a significant interaction between housing and stroke groups or a significant main effect of either stroke or housing condition.

Name of the Gene	Interaction (Housing x Surgery)	Main effect of Housing	Main effect of Stroke	Up or Down (vs. PH)
PSD95	NS	NS	0.0119	↑
Gabra5	0.0004	NS	0.0001	↑
Gria2	0.0224	NS	0.0001	↑
Grin2b	NS	0.0002	0.0002	↑
Grin2c	0.0138	0.0248	0.0003	↑
Mapk1	NS	0.0023	0.0758	↓
BDNF	0.0001	.0007	0.0001	↓
IGF1	NS	0.0425	0.0156	↓
CamK2a	NS	0.0048	0.002	↑
Ppp3ca	0.0005	0.0215	0.0001	↑
Rela	NS	NS	0.0064	↑
Nfkb1	0.0179	0.0442	0.0249	↑
Tnf	NS	0.0225	0.004	↓
