# Supplementary material for: Reversal of the Detrimental Effects of Post-Stroke Social Isolation by Pair-Housing is Mediated by Activation of BDNF-MAPK/ERK in Aged Mice
Source: Sci Rep. 2016 Apr 29;6:25176. doi: 10.1038/srep25176 (PMC4850427; doi:10.1038/srep25176)
Supplement: Supplementary Information [file srep25176-s1.pdf]

Supplementary informations:

Reversal of the Detrimental Effects of Post-Stroke Social Isolation by Pair-Housing is Mediated by Activation of BDNF-MAPK/ERK in Aged Mice

Rajkumar Verma[a], Nia M. Harris[a], Brett D Friedler[a], Joshua Crapser[a], Anita R Patel[a], Venugopal Venna[a] & Louise D McCullough[a,b,*]

[a] Department of Neuroscience, University of Connecticut Health Center, Farmington, CT, USA

[b] Department of Neurology, University of Texas Health Science Center, Houston, TX 77030

Running Title: Isolation exacerbates stroke in aged mice

Corresponding author: Louise D. McCullough,

University of Texas Health Science Center, McGovern Medical School

6431 Fannin, Houston, TX 77030 Phone: 713-500-7079

E-mail: Louise.D.McCullough@uth.tmc.edu

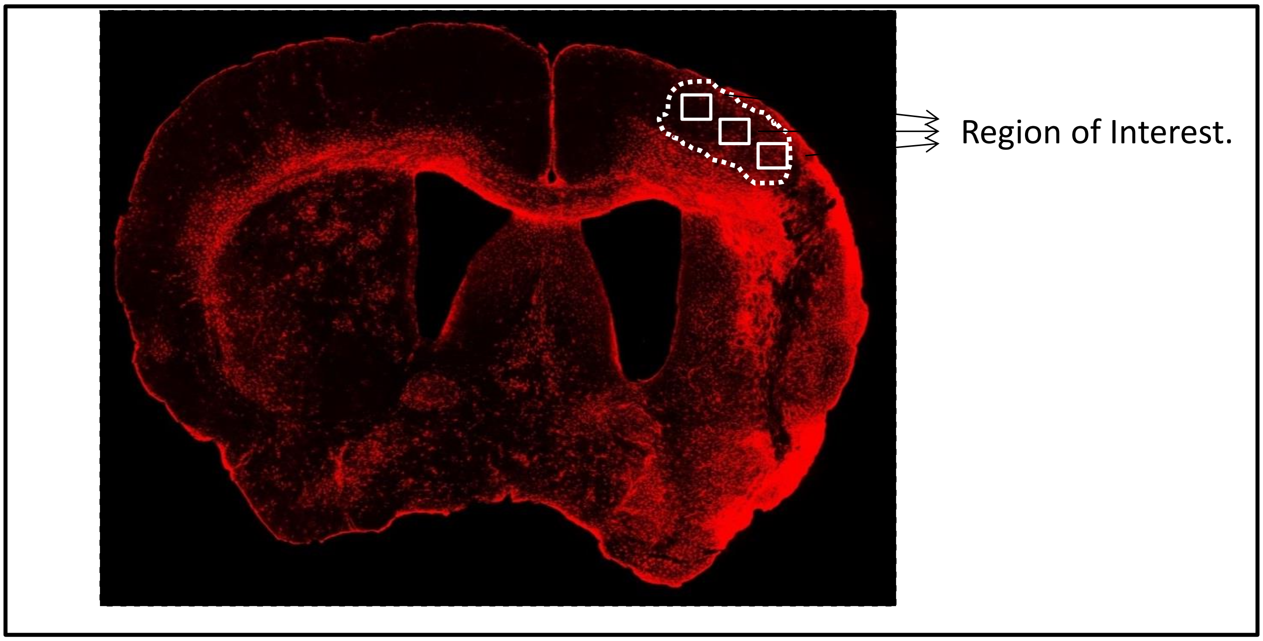

Supplementary Figure 1: Illustration showing the "region of interest" for IHC works.

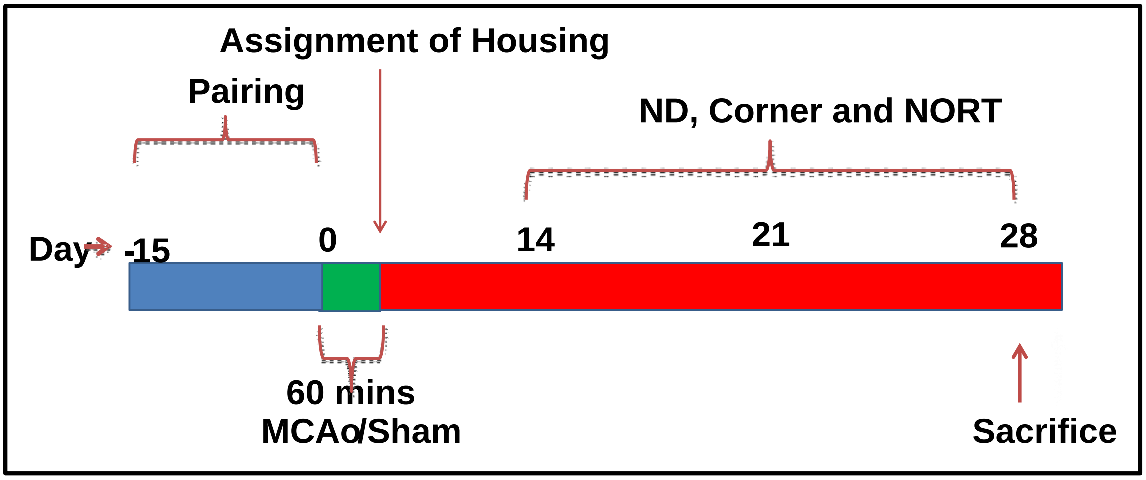

Supplementary Figure 2: Schematic of the behavioral testing paradigm in the chronic survival group.
